# Supplementary material for: MetaRibo-Seq measures translation in microbiomes
Source: Nat Commun. 2020 Jun 29;11:3268. doi: 10.1038/s41467-020-17081-z (PMC7324362; doi:10.1038/s41467-020-17081-z)
Supplement: Supplementary file 10 — Supplementary Data 7 [file 41467_2020_17081_MOESM10_ESM.zip › File2/Confidence_High_Taxonomy/201596_out.krona.html]

Javascript must be enabled to view this page.

members
magnitude
magnitudeUnassigned
count
unassigned
taxon
rank

201596\_out

5


SRS023914\_contig\_number\_contig-100\_5094.126139
1
superkingdom
2

2759
2
superkingdom

2
kingdom
4751

2
subkingdom
451864

phylum
2
4890

147538
subphylum
2

1

SRS098571\_contig\_number\_54450
147550
2
class

1
subclass
222543

1
order
5125

2502719
family
1

1
genus
284134

species
1
284135

SRS051031\_contig\_number\_35517


SRS013687\_contig\_number\_contig-100\_2415.123128SRS1041145\_contig\_number\_32492
2
